# Precise Characterization and Tracking of Stably Inherited Artificial Minichromosomes Made by Telomere-Mediated Chromosome Truncation in *Brassica napus*

**DOI:** 10.3389/fpls.2021.743792

**Published:** 2021-10-04

**Authors:** Xiangzhen Yin, Yingxin Zhang, Yuhong Chen, Jingqiao Wang, Richard R.-C. Wang, Chengming Fan, Zanmin Hu

**Affiliations:** ^1^State Key Laboratory of Plant Cell and Chromosome Engineering, Institute of Genetics and Developmental Biology, Innovation Academy for Seed Design, Chinese Academy of Sciences, Beijing, China; ^2^College of Agriculture, Yangtze University, Jingzhou, China; ^3^Institute of Economical Crops, Yunnan Agricultural Academy, Kunming, China; ^4^Forage and Range Research Laboratory, United States Department of Agriculture, Agricultural Research Service, Utah State University, Logan, UT, United States; ^5^College of Agriculture, University of Chinese Academy of Sciences, Beijing, China

**Keywords:** *Brassica napus*, telomere, chromosome truncation, minichromosome, genome resequencing, insertion site-specific PCR, multi-FISH

## Abstract

Plant artificial minichromosomes are the next-generation technology for plant genetic engineering and represent an independent platform for expressing foreign genes and the tools for studying the structure and function of chromosomes. Minichromosomes have been successfully produced by telomere-mediated chromosome truncation in several plants. However, previous studies have primarily focused on the construction and rough characterization of minichromosomes, while the development of stably inherited minichromosomes and their precise characterization and tracking over different generations have rarely been demonstrated. In this study, a 0.35-kb direct repeat of the *Arabidopsis* telomeric sequence was transformed into *Brassica napus* to produce artificial minichromosomes, which were analyzed by multifluorescence *in situ* hybridization (multi-FISH), Southern hybridization, and primer extension telomere rapid amplification (PETRA). The stably inherited minichromosomes C2 and C4 were developed by crossing transgenic plants with wild-type plants and then selfing the hybrids. Notably, two truncation sites on chromosomes C2 and C4, respectively, were identified by resequencing; thus, the artificial minichromosomes were tracked over different generations with insertion site-specific PCR. This study provided two stably inherited minichromosomes in oilseed rape and describes approaches to precisely characterize the truncation position and track the minichromosomes in offspring through multi-FISH, genome resequencing, and insertion site-specific PCR.

## Introduction

Genetic engineering enables the reduction of the cost of weed and pest control and the improvement of the quality and productivity of many crops. To date, most genetic engineering for complex or combined traits requires the simultaneous expression of multiple genes. However, in traditional technology employed for genetic engineering, only one or several genes can be transformed at a time and integrated in the genic region of a chromosome at random (Houben and Schubert, 2007; Yu et al., 2007b). Artificial minichromosomes can be used as independent platforms for stacking multiple foreign genes without gene segregation (Birchler et al., 2016). Artificial minichromosomes can also help to answer fundamental questions regarding chromosomal structure and function (Houben and Schubert, 2007; Yu et al., 2007b).

There are two approaches to construct artificial chromosomes: bottom-up approach and top-down approach (Houben and Schubert, 2007). The bottom-up approach has been successfully utilized to produce human artificial chromosomes (HACs) by introducing cloned centromeric and telomeric DNA into human cultured cells to form *de novo* HACs ranging in size from 1 to 10 Mb (Harrington et al., 1997; Ikeno et al., 1998; Henning et al., 1999; Ebersole et al., 2000; Grimes et al., 2001; Mejia et al., 2001). However, in plants this approach is unlikely to work because there is an epigenetic basis for centromere function (Birchler et al., 2016).

In the top-down approach, telomere-associated chromosome truncation is employed to produce minichromosomes; this approach has been applied successfully in mammals and plants. In mammals, human telomeric sequences were introduced into mammalian cells, and the distal portion of an autonomous chromosome could be truncated by the formation of a new telomere at the integration site (Farr et al., 1991). In plants, minichromosomes from autonomous chromosomes can be successfully produced by transforming constructs with telomeric sequences into maize (Yu et al., 2006, 2007a), *Arabidopsis* (Nelson et al., 2011; Teo et al., 2011), barley (Kapusi et al., 2012), rice (Xu et al., 2012), common wheat (Yuan et al., 2017), and oilseed rape (Yan et al., 2017). Therefore, telomere-associated chromosome truncation as a top-down approach to generate artificial chromosomes is promising in plants.

The characterization of chromosome truncation is an important step in the generation of artificial minichromosomes through telomere-mediated chromosome truncation. At present, the methods of identifying minichromosomes made by telomere-mediated chromosome truncation include (1) fluorescence *in situ* hybridization (FISH); (2) Southern blot analysis; and (3) primer extension telomere repeat amplification (PETRA) (Heacock et al., 2004). FISH is helpful for demonstrating the truncations of large chromosomes, but difficult to effectively characterize the truncations of small chromosomes. Due to highly condensed metaphase chromosomes, FISH was not always effective in distinguishing whether chromosomal truncation occurred when a transgene was at the distal chromosomal position (Yu et al., 2006). Southern blotting can be employed to demonstrate new telomere formation in minichromosomes as a smear if DNA is digested with a restriction enzyme that cleaves a nearby internal site (Yu et al., 2006; Yan et al., 2017). PETRA can detect newly produced telomeres and their sizes (Nelson et al., 2011; Teo et al., 2011; Yan et al., 2017). If multisite insertions were to occur in one transgenic transformant, these three methods would have limited ability to accurately identify the truncated sites and the chromosomes from which artificial chromosomes were derived. To date, the development of stably inherited minichromosomes, the precise characterization of their truncation positions and the tracking of minichromosomes over different generations have rarely been reported.

*Brassica napus* (oilseed rape), a natural allotetraploid (Uagaharu, 1935), is one of the most economically important oil crops in the world. The chromosomes of *B. napus* are small. In a previous study, minichromosomes were developed using telomere-mediated chromosomal truncation in *B. napus* (Yan et al., 2017). However, the precise characterization of truncated chromosomes and the accurate tracking of minichromosomes across different generations need to be improved considerably. In this report, we constructed the minichromosomes C2 and C4 in *B. napus* using the vector pWY86-1 with 0.35-kb *Arabidopsis* telomere repeats. We characterized chromosome truncations and new telomere formation in transgenic plants by a combination of multi-FISH, Southern blot analysis and PETRA. Furthermore, we identified two truncation positions on chromosomes C2 and C4 through genome resequencing and accurately tracked the stable inheritance of minichromosomes in the offspring of transgenic plants through insertion site-specific PCR. We also showed that the exogenous gene could be integrated into the minichromosomes by site-specific recombination using Cre. In this study, we generated the stably inherited minichromosomes C2 and C4 in oilseed rape, and described approaches to precisely characterize the positions of chromosome truncations mediated by telomere and to track the minichromosomes in the offspring.

## Materials and Methods

### Plant Expression Vectors

Vectors pWY86, pWY96 and pCre1301 were generously provided by Dr. James A. Birchler. One 0.35-kb TRA (telomere repeat array) derivative of pWY86 was obtained by transforming pWY86 into Stbl4 cells (Invitrogen) and screening individual colonies by restriction digestion with *Eco*RI and *Bam*HI and renamed pWY86-1. As reported previously, pWY96 (Yu et al., 2006; Yan et al., 2017), including the elements common to pWY86-1 and the Pmas-HPT-Tmas gene-expression cassette replacing the 0.35-kb telomere sequence, was used as a FISH probe to detect all transgenes from pWY86-1 transformations. pCre1301 contained a P35S-lox66-Cre-Tnos cassette and was used to demonstrate the utility of minichromosome platforms by a site-specific recombination system. P35S-HPT-T35S and P35S-GUS-Tnos gene expression cassettes were selection marker genes for pCre1301 transformation. In particular, the pWY86-1 construct was propagated in Stbl4 and grown at 30°C instead of 37°C for the maintenance of repetitive telomere sequences.

### Gene Transformation

The final binary vectors pWY86-1 and pCre1301 were transferred into *A. tumefaciens* strain GV3101 by the freeze-thaw method (Holsters et al., 1978). In particular, the integrity of the pWY86-1 construct in *A. tumefaciens* was confirmed by extracting plasmids from a sample of the cells used for *B. napus* transformation and analyzing by restriction digestion. *B. napus* var. Westar was transformed using hypocotyl explants by the modified method of Deblock et al. (1989). *B. napus* var. Westar seeds were soaked in 1‰ HgCl_2_ for 10 min, rinsed with aseptic water three times, and then incubated without light on hormone-free MS medium for 7 days. PPT (10 mg/L) and kanamycin (25 mg/L) were used to select transformed plantlets of pWY86-1 and pCre1301, respectively. Finally, kanamycin/bar-resistant plantlets were transferred to pots and grown in a greenhouse.

### Screening of Transgenic Plants and Histochemical GUS Assay

Fresh leaf tissue was collected and quickly frozen in liquid nitrogen, and genomic DNA was extracted by the cetyltrimethylammonium bromide (CTAB) method (Murray and Thompson, 1980). PCR-based screening was employed to detect the presence of the transgenic elements. Specific primers and the sizes of the expected PCR products are summarized in Supplementary Table 1. For amplification with Taq DNA polymerase, DNA was denatured at 95°C for 2 min, followed by 30 cycles of 30 s at 95°C, 30 s at annealing temperature and 60–120 s at 72°C with a final extension at 72°C for 7 min. The PCR products were analyzed by agarose gel electrophoresis. For the detection of pCre1301 transgenic plants and the hybrid progenies achieved by crossing pWY86-1 transgenic plants with pCre1301 transformants, the GUS *uidA* gene expression test was carried out with GUS stain solution (38/62 mM NaH_2_PO_4_/Na_2_HPO_4_ pH 7.0, 10 mM Na_2_EDTA, 0.5 mM K_4_Fe(CN)_6_, 0.5 mM K_3_Fe(CN)_6_, 0.1% Triton X-100, 500 μg/mL X-Gluc). Leaves were stained with GUS solution at 37°C overnight and fixed with 70% ethanol.

### Fluorescence *in situ* Hybridization

Root tips and flower buds of pWY86-1 transformants and their progenies were collected to perform chromosome preparations according to the method by Zhang et al. (2016). FISH was performed in accordance with the method described by Kato et al. (2004). The plasmid pWY96 containing the elements common to pWY86-1 but without the 0.35-kb telomere sequence was used as a FISH probe to detect all transgenes in the pWY86-1 transformants. 5S rDNA, 45S rDNA, BAC clones KBrB072L17, and CentBr1 (Xiong and Pires, 2011) were used as FISH probes to localize the positions of transgenes and the truncation of a specific chromosome and to observe the behavior of minichromosomes in meiosis. Probe DNAs were labeled with Texas Red-5-dCTP (Perkin Elmer Life Sciences) or ChromaTide™ Alexa Fluor™ 488-5-UTP (Invitrogen) by nick translation. FISH images were photographed with an epifluorescence Olympus BX61 microscope equipped with a cooled charge-coupled device camera and MetaMorph software (Han et al., 2009).

### Southern Blotting

Genomic DNA of transgenic plants was isolated by the CTAB method (Murray and Thompson, 1980). Thirty micrograms of genomic DNA from each sample was digested with 100 units of restriction enzyme *Hin*dIII or *Eco*RV (New England Biolabs) in a 30-μl volume at 37°C overnight, fractionated in a 0.8% agarose gel at 25 volts overnight, denatured, and transferred to an Amersham Hybond N+ nylon membrane (GE). *FLP* was used as the probe and was ^[32P]^dCTP-labeled in accordance with the Random Primer DNA Labeling Kit (TaKaRa). DNA hybridization with ^[32P]^dCTP-labeled probes and X-ray autoradiography were conducted according to the method of Sambrook and Russell (2001).

### Primer Extension Telomere Repeat Amplification

PETRA was performed as described previously (Heacock et al., 2004; Nelson et al., 2011). The PETRA-T adapter was annealed to the 3′ G-rich overhang of the newly formed telomere and extended by DNA polymerase I. Then, the PETRA-A reaction was carried out using the PETRA-A primer complementary to primer PETRA-T and either of two primers specific to pWY86-1, p1 and p4 (Supplementary Table 1). The PCR products were electrophoretically separated, transferred to a nylon membrane and hybridized with the ^32^P-labeled pWY96 probe.

### Genome Resequencing Analyses

Genomic DNA was extracted from the leaves of three progeny (86-1-75-10-49, 86-1-75-10-74, and 86-1-75-10-80) of transgenic plants 86-1-75 by the CTAB method (Murray and Thompson, 1980). At least 6 μg of genomic DNA from each sample was used to construct a sequencing library following the manufacturer's instructions (Illumina Inc.). Paired-end sequencing libraries with an insert size of ~150 bp were sequenced on an Illumina HiSeq 2000 sequencer. Each sample produced 40 Gb granting Q30 up to 80%. The potential insertion sites were detected by genome resequencing analyses performed by mapping to the *B. napus* genome (www.ncbi.nlm.nih.gov/assembly/GCF_000686985.2/; Bra_napus_v2.0) and the sequence between LB and RB of pWY86-1.

### Identification of Chromosome Truncations by Insertion Site-Specific PCR

Five pair primers (Supplementary Table 1) were designed according to the flanking sequences of the five putative insertion sites. The primers near LB of pWY86-1 were named forward primers (A/Cn_1F), and the other primers near RB of pWY86-1 were annotated as reverse primers (A/Cn_1R). Blp_F (Supplementary Table 1) was paired with A/Cn_1F, and p4 was paired with A/Cn_1R. For detection of insertion sites, if no insertion was anchored on the targeted chromosome, no bands could be observed using both Blp_F to A/Cn_1F and p4 to A/Cn_1R; if one insertion was anchored on the targeted chromosome and no chromosome truncation occurred, one band should be achieved using Blp_F to A/Cn_1F and p4 to A/Cn_1R, respectively; if one insertion led to the targeted chromosome truncation, one band should be observed using Blp_F to A/Cn_1F but no band using p4 to A/Cn_1R.

### Detection of Pollen Fertility and Activity

FDA-PI was used to detect the fertility and activity of pollens with a fluorescence microscope using the following description method. Dissolve 10 mg FDA and 5 mg PI in a small amount of acetone and then add H_2_O to a volume of 5 mL. The solution of FDA-PI was stored at 4°C without light. Add proper amounts of pollens in the morning and 100 μL FDA-PI solution to the bottom of the centrifuge tube and then mix at low speed with a vortex meter. Ten microliters of the mixture was dropped on a clean slide after 5 min of staining and then observed by an Olympus BX61 fluorescence microscope. Green fluorescent pollen was active, and red fluorescent pollen was inactive (Jones and Senft, 1985; Oliver et al., 2005).

## Results

### Plasmid Selection and *B. napus* Transformation

**Figure 1:**
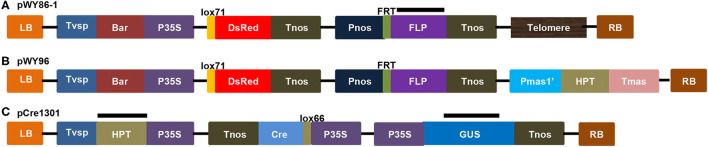
Constructs of pWY86-1, pWY96, and pCre1301. **(A)** Construct pWY86-1 derived from pWY86 (Yu et al., 2006), containing *Arabidopsis* telomeric sequence and promoter-less lox71-DsRed. **(B)** The contrasting construct pWY96 (Yu et al., 2006) was used as a FISH probe to detect transgenes from pWY86-1 transformations. **(C)** Construction of pCre1301 containing the P35S-lox66-Cre-Tnos expression cassette. The screening marker genes in plants are *HPT* and *GUS*. LB, T-DNA left border; RB, T-DNA right border; Tvsp, terminator from soybean vegetative storage protein gene; Bar, bialaphos resistance gene as a selection marker gene; P35S, 35S promoter from cauliflower mosaic virus; Tnos, Nos terminator from *Agrobacterium*; Tmas, Mas terminator from *Agrobacterium*; Pnos, Nos promoter from *Agrobacterium*; Pmas1', Mas promoter from *Agrobacterium*; lox and FRT, site-specific recombination sites; HPT, hygromycin B-resistance gene as a selection marker gene; DsRed, red fluorescent protein gene; FLP, recombinase gene; Cre, recombinase gene; GUS, *uidA* gene; lox71, the lox71 site; lox66, the lox66 site; Telomere, telomere units of pAtT4 isolated from *Arabidopsis thaliana*. The black bars showed the targeted region by PCR for checking T-DNA integrity using primers FLP_F, FLP_R, GUS_F, GUS_R, HPT_F and HPT_R (Supplementary Figure 1 and Supplementary Table 1).

pWY86-1 (Figure 1A) bearing a 0.35-kb direct repeat of *Arabidopsis* telomeric sequence was derived from pWY86 (Yu et al., 2006). An *Arabidopsis* telomeric sequence and promoter-less lox71-DsRed were included to cause chromosomal truncations and to determine the recombination of foreign genes into the minichromosome, respectively. A Pnos-FRT-FLP-Tnos gene-expression cassette was used for potential manipulations using the FRT-FLP site-specific recombination system. The P35S-Bar-Tvsp gene-expression cassette was included as a selection marker gene for *B. napus* transformation. As reported previously, pWY96 (Yu et al., 2006; Yan et al., 2017) in Figure 1B, which included the elements common to pWY86-1 and the Pmas-HPT-Tmas gene-expression cassette replacing the units 0.35 telomere sequence, was used as a FISH probe to detect all transgenes from pWY86-1 transformations. pCre1301 (Figure 1C) containing a P35S-lox66-Cre-Tnos cassette was utilized to demonstrate the utility of minichromosome platforms by a site-specific recombination system. P35S-HPT-T35S and P35S-GUS-Tnos gene expression cassettes were selection marker genes for pCre1301 transformation. pWY86-1 and pCre1301 were used to transform *B. napus* var. Westar hypocotyls by an *Agrobacterium*-mediated gene transformation method. In total, seven pWY86-1 transgenic plants and four pCre1301 transgenic plants were obtained. PCR screening of pWY86-1 transgenic plants was performed by amplifying the FLP gene fragment (1,115 bp), with the construct pWY86-1 serving as a positive control and WT (wild-type Westar) serving as a negative control. The results (Supplementary Figure 1A) showed that only three (86-1-73, 86-1-75, and 86-1-90) of seven pWY86-1-transformed plants were positive, suggesting that pWY86-1 had been integrated into the oilseed rape genome. The four pCre1301 transgenic plants (1301-1, 1301-2, 1301-3, and 1301-4) were identified by PCR targeting the *Hyg* and *GUS* genes (Supplementary Figure 1B).

### Determination of Chromosomal Truncations in *B. napus* Transgenic Lines

**Figure 2:**
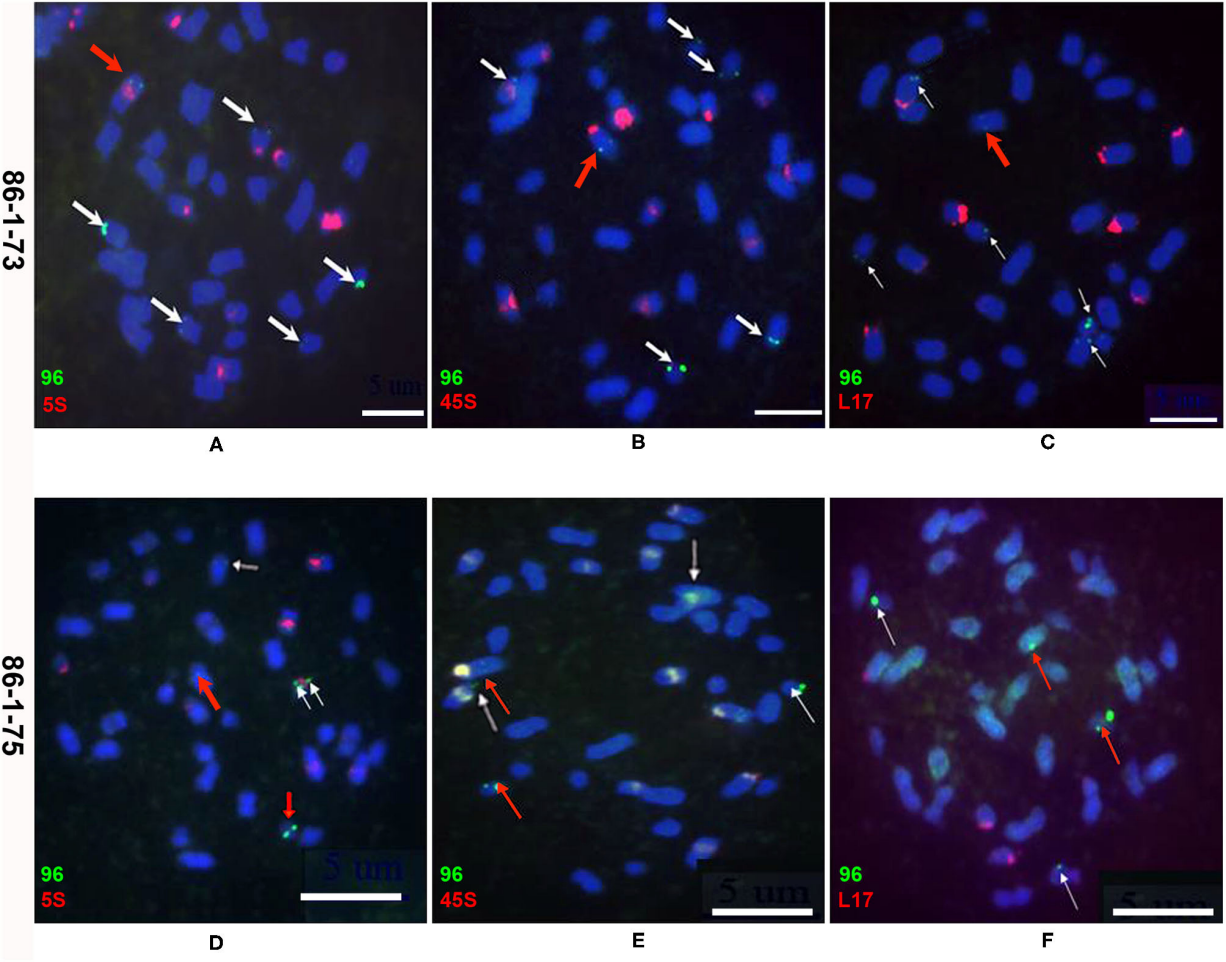
Molecular cytogenetics analysis of chromosome truncations in transgenic plants 86-1-73 and 86-1-75 by FISH. Metaphase chromosomes of root tips were hybridized with a pWY96 probe (96, green). Arrows indicate the chromosomes and the sites of transgene insertions. Scale bar = 5 μm. **(A–C)** Six transgenic sites were observed with five distal loci in transgenic plant 86-1-73; **(D–F)** Five transgenic sites were observed with three distal loci in transgenic plant 86-1-75; pWY96 **(A–F)** was labeled with Alexa Fluor™ 488-5-UTP (green); 5S (5S rDNA; **A,D**), 45S (45S rDNA; **B,E**) and KBrB072L17 (L17, **C,F**) were labeled with Texas Red-5-dCTP (red). White arrows indicated distal loci and red arrows indicated internal loci.

To detect chromosomal truncations, metaphase chromosomes from the root tips of two transgenic plants (86-1-73 and 86-1-75) and their progeny were probed by FISH with a pWY96 probe (Yu et al., 2006). The results showed that numerous transgene loci were observed in the two transformed plants. Six transgenic sites containing five distal loci were observed in transgenic plant 86-1-73 (Figures 2A–C and Table 1), and five transgenic sites including three distal loci were observed in transgenic plant 86-1-75 (Figures 2D–F and Table 1). We tried to identify which chromosomes were truncated using 5S rDNA, 45S rDNA, and a *B. rapa* BAC clone KBrB072L17 according to Xiong and Pires (2011), but failed in T0 transformed plants (Figure 2). Nevertheless, because *B. napus* chromosomes are notably small, it was difficult to use molecular cytogenetics analysis to clearly determine whether chromosomal truncations occurred.

**Table 1:** The locus number for the two transgenic plants, 86-1-73 and 86-1-75.

**Transgenic plants**	**Transgene loci**
86-1-73	Five distal loci, one internal loci
86-1-75	Three distal loci, two internal loci

**Figure 3:**
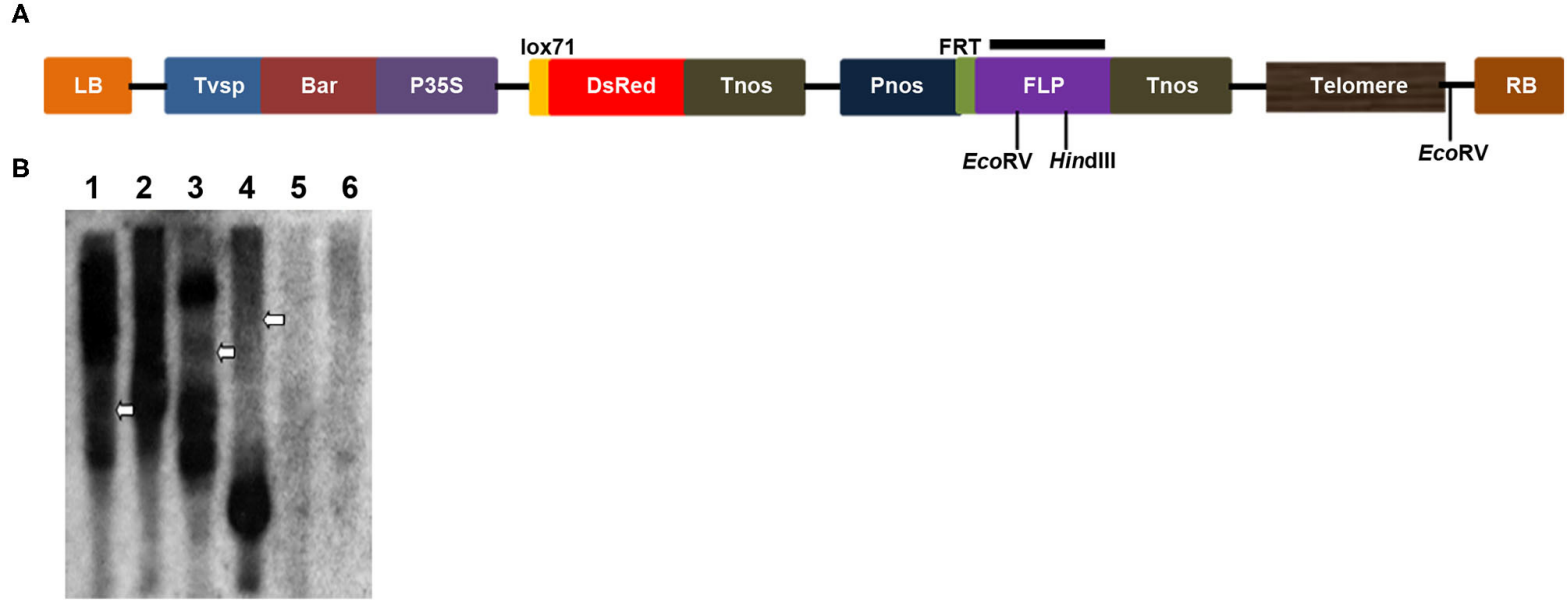
Chromosomal truncations demonstrated by a Southern blot analysis with enzymatic digestions. **(A)** Scheme of the T-DNA region of the pWY86-1 construct with restriction sites used in DNA gel blot analysis. Black bar shows the region targeted by hybridizing *FLP* probes. **(B)** Restriction mapping of two transgenes 86-1-73 and 86-1-75 by a Southern blot. Genomic DNAs of lanes 1, 3, and 5 were digested with *Hin*dIII and lanes 2, 4, 6 digested with *Eco*RV, respectively. Genomic DNAs of lanes 1 and 2 were from 86-1-73, lanes 3 and 4 from 86-1-75, and lanes 5 and 6 from WT. The Southern blot was hybridized with the ^32^P-labeled *FLP* probe. Telomere smears are indicated by arrowheads.

Because of the limitations of the molecular cytogenetics analysis, Southern blotting was performed to characterize potential chromosomal truncations (Yu et al., 2006; Teo et al., 2011; Yan et al., 2017). Chromosomal truncations result in the formation of new telomeres at broken ends to maintain chromosome stability, and new telomeres exhibit different sizes in different cells; therefore, a smear band of heterogeneous telomeres in a Southern blot will be observed if DNA is digested with a restriction enzyme that cleaves a nearby internal site (Farr et al., 1991; Yu et al., 2006). In this study, genomic DNA from two transgenic plants, 86-1-73 and 86-1-75, was digested with *Hin*dIII or *Eco*RV, and a Southern blot was hybridized with a ^32^P-labeled *FLP* gene fragment. Smears were observed for both transgenic plants (Figure 3), which suggested that chromosomal truncations occurred in the two transgenic lines.

**Figure 4:**
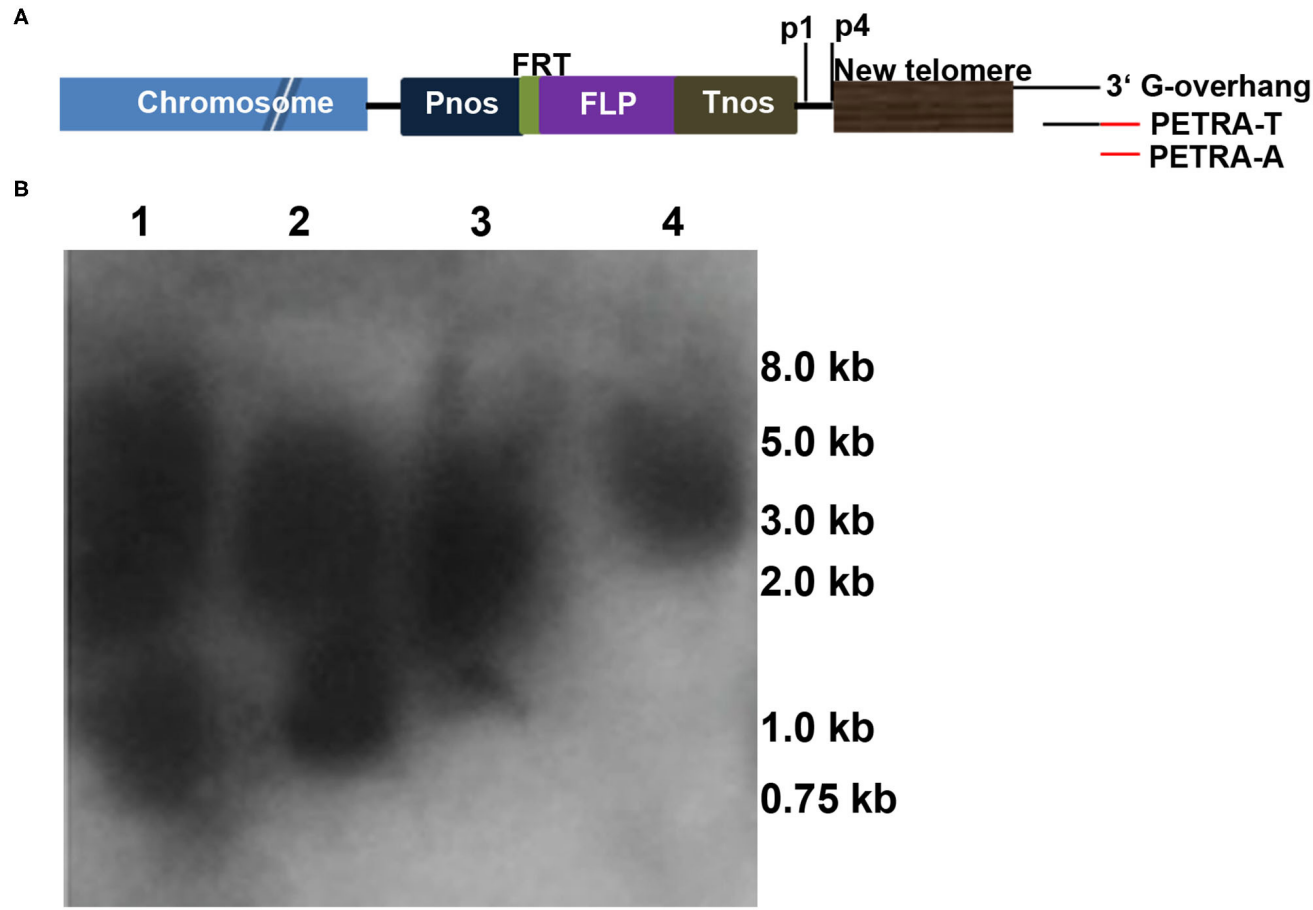
Chromosomal truncations revealed by PETRA. **(A)** Schematic diagram depicting the PETRA assay. PETRA-T oligonucleotides (Supplementary Table 1) were annealed to the G-rich overhang at the 3′ end of the *de novo* telomeres, and primer extension was performed with DNA polymerase I. Then, the primer extension products were subjected to PCR amplification using the transgene-specific primer p1/p4 (Supplementary Table 1) and the PETRA-T complementary primer PETRA-A (Supplementary Table 1). The resulting PCR products were separated by gel electrophoresis, transferred to membranes and hybridized with the ^32^P-labeled pWY96 probe. **(B)** PETRA reactions with primers p1 and p4 were performed for each of the two transgenes, 86-1-73 and 86-1-75. Lane 1: p1, 86-1-75; lane 2: p4, 86-1-75; lane 3: p1, 86-1-73; lane 4: p4, 86-1-73. M, Trans2K PlusII DNA Marker. Lanes 1–4 show the staggered size products.

In addition to FISH and Southern blot analyses, PETRA (Heacock et al., 2004) was performed to identify new telomere formation (Nelson et al., 2011; Yan et al., 2017) in this study as follows. The PETRA-T adapter (Figure 4A and Supplementary Table 1) was annealed to the 3' G-rich overhang of the new telomeres and extended by DNA polymerase I to form a double-stranded joint at the ends of the telomeres. Then, the PETRA-A reaction was performed using the PETRA-A primer (Figure 4A and Supplementary Table 1) complementary to PETRA-T, and the reverse primer (p1 or p4; Figure 4A and Supplementary Table 1) bound a specific sequence adjacent to the telomere sequence in the pWY86-1 construct. A smear band is detected if a new telomere formation occurs due to telomere-mediated chromosomal truncation. To detect the chromosomal truncations in 86-1-73 and 86-1-75, PETRA reactions were performed and smears were observed in the two transgenic plants (Figure 4B). The result confirmed *de novo* telomere formation due to chromosomal truncation, which suggested minichromosomes were successfully made by telomere-mediated chromosome truncations in 86-1-73 and 86-1-75.

### Characterization of the Truncation Positions by Genome Resequencing Analysis and Insertion Site-Specific PCR

**Figure 5:**
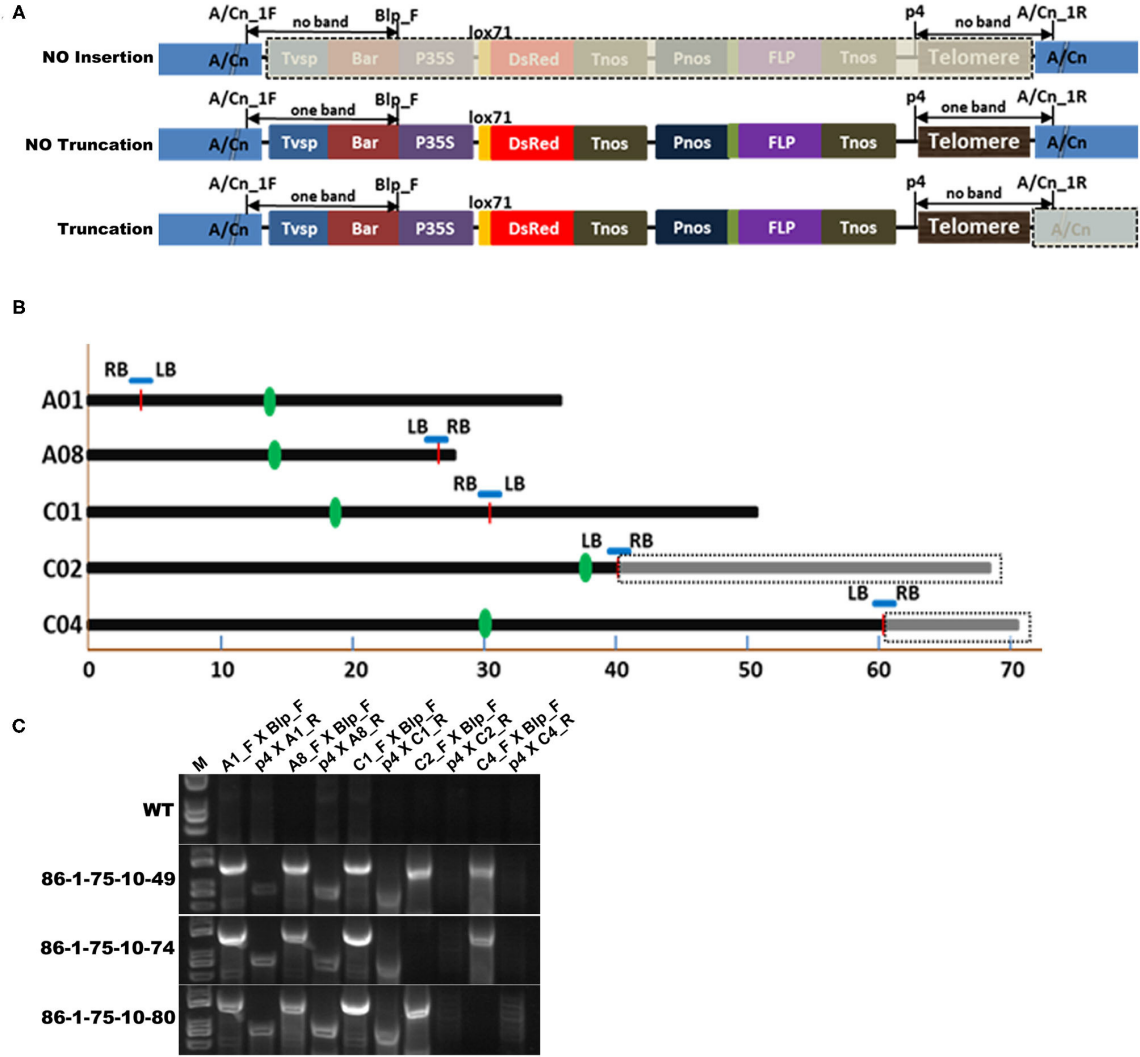
Confirmation of truncation positions by genome resequencing analysis and insertion site-specific PCR. **(A)** Schematic diagram depicting confirmation of the truncation on chromosomes by insertion site-specific PCR. A/Cn indicate the chromosome An or Cn. The forward primer binding to the flanking sequence near LB of pWY86-1 of the potential insertion site was A/Cn_F. And the reverse primer binding to the flanking sequence near RB of pWY86-1 was A/Cn_R. Blp_F (Supplementary Table 1) was paired with A/Cn_F, and p4 (Figure 4 and Supplementary Table 1) was paired with A/Cn_R. When there was no insertion on chromosome A/Cn, no bands occurred using the two primer pairs (Blp_F with A/Cn_F and p4 with A/Cn_R); when there was one insertion but no chromosome truncation, one band occurred using the two primer pairs; when there was one chromosome truncation event, one band occurred using primer pairs Blp_F and A/Cn_F, but no band occurred using primer pairs p4 and A/Cn_R. **(B)** Five potential insertion sites in 86-1-75 by genome resequencing analysis according to the reference genome Bra_napus_v2.0 (https://www.ncbi.nlm.nih.gov/assembly/GCF_000686985.2/). Three progeny (86-1-75-10-49, 86-1-75-10-74, and 86-1-75-10-80; Table 2) of 86-1-75 were genome-resequenced. The five insertion sites were distributed on chromosomes A1, A8, C1, C2, and C4 of 86-1-75. The insertion sites were indicated by red short lines. The truncations of C2 and C4 were indicated by dashed box. The centromeric positions were indicated by green bar. **(C)** Confirmation of the truncation on chromosomes by PCR. According to **(A)**, five primer pairs (A1_F/A1_R, A8_F/A8_R, C1_F/C1_R, C2_F/C2_R, and C4_F/C4_R; Supplementary Table 1) were designed and PCR was conducted. The results showed the insertions on chromosomes C4 of 86-1-75-10-74 and C2 of 86-1-75-10-80 led to chromosome truncations as well as the two insertions on chromosomes C2 and C4 of 86-1-75-10-49, respectively.

Because FISH, Southern blotting and PETRA have limitations in accurately identifying the sites of telomere-mediated chromosomal truncations and the chromosomes from which the minichromosomes by telomere-mediated chromosome truncations are derived, we introduced resequencing combined with insertion site-specific PCR (Figure 5) to confirm minichromosomes. The genome of *B. napus* has been sequenced (Chalhoub et al., 2014); therefore, the potential insertion sites of the chromosomes can be quickly and comprehensively identified by resequencing. In this study, the genomes of three progenies of 86-1-75 (i.e., 86-1-75-10-49, 86-1-75-10-74, and 86-1-75-10-80) were selected to be resequenced and subsequently analyzed by mapping to the *B. napus* genome (www.ncbi.nlm.nih.gov/assembly/GCF_000686985.2/; Bra_napus_v2.0). The results showed that 86-1-75-10-49 had five insertion sites distributed on chromosomes A1, A8, C1, C2, and C4, 86-1-75-10-74 had four insertion sites distributed on chromosomes A1, A8, C1, and C4, and 86-1-75-10-80 had four insertion sites distributed on chromosomes A1, A8, C1, and C2 (Figures 5B,C and Table 2). Then, five pairs of primers (A1_F/A1_R, A8_F/A8_R, C1_F/C1_R, C2_F/C2_R and C4_F/C4_R; Supplementary Table 1) were designed according to the flanking sequences of the five insertion sites (Figure 5B), and PCR was subsequently performed to confirm the sites of truncations on chromosomes according to the design of detection sites in Figure 5A. The PCR results (Figure 5C) showed as follows. For all three detected plants, one band resulted when primer pair combinations of Blp_F with A1_F/A8_F/C1_F, or p4 with A1_R/A8_R/C1_R were used. This indicated that there was insertion in A1, A8, and C1 chromosomes without truncation. In 86-1-75-10-49, one band occurred using the primer pairs of Blp_F and C2/C4_F, but no band using p4 and C2/4_R combination. It indicated that there was truncation on chromosomes C2 and C4. In 86-1-75-10-74, one band resulted using Blp_F and C4_F, but no band using p4 and C4_R. It indicated that there was truncation on Chromosome 4. In 86-1-75-10-80, one band occurred using primer pairs of Blp_F and C2_F, but no band using p4 and C2_R. It indicated there was truncation on Chromosome 2. These PCR results indicated that the insertions on chromosomes C4 of 86-1-75-10-74 and C2 of 86-1-75-10-80 led to chromosome truncations, as well as the two insertions on chromosomes C2 and C4 of 86-1-75-10-49, and that the insertions on chromosomes A1, A8, and C1 did not result in chromosome truncations. On the basis of these results, the primers C2_F/C2_R and C4_F/C4_R combined with Blp_F and p4 for identifying the truncated C2 and C4, respectively, can be employed to track the minichromosomes made by telomere-mediated chromosome truncations in the offspring of the subsequent generations.

**Table 2:** Potential insertion sites in three progeny of 86-1-75 by genome resequencing analysis.

**Chr**.	**Chr. length**	**Insert position**	**85-1-75-10-49**	**85-1-75-10-74**	**85-1-75-10-80**
A1	35,822,559	3,856,908	+	+	+
A8	27,741,901	26,437,963	+	+	+
C1	50,776,417	30,422,504	+	+	+
C2	68,396,429	40,113,412	+	–	+
C4	70,552,672	60,231,159	+	+	–

*The potential insertion sites in the three progeny (86-1-75-10-49, 86-1-75-10-74, and 86-1-75-10-80) of 86-1-75 by genome resequencing analysis according to the reference genome Bra_napus_v2.0 (https://www.ncbi.nlm.nih.gov/assembly/GCF_000686985.2/). There were five potential insertion sites distributed on chromosomes A1, A8, C1, C2, and C4 in 86-1-75-10-49, four potential insertion sites distributed on chromosomes A1, A8, C1, and C2 in 86-1-75-10-74, and four potential insertion sites distributed on chromosomes A1, A8, C1, and C4 in 86-1-75-10-80*.

**Figure 6:**
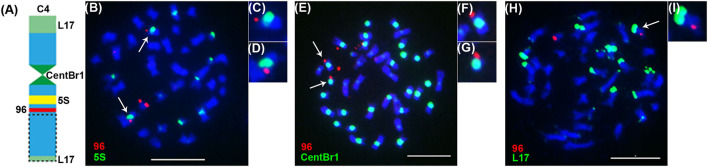
Detection of the truncated chromosome C4 in 86-1-75-10-74 by multi-FISH. **(A)** The features of chromosome C4 in *B. napus* (Xiong and Pires, 2011) and the site hybridized by pWY96 probe (red). 5S rDNA (5S; yellow) was located on the long arm of chromosome C4 near the centromere, a strong and a weak signals of BAC clone KBrB072L17 (L17; pale green) were located on chromosome C4 at the end of its short and long arms, respectively, and CentBr1 (green) repeats could be visualized in *B. napus* chromosome C4 (Xiong and Pires, 2011). **(B,E,H)** were hybridized with the labeled pWY96 probe, and they were also hybridized with the labeled 5S, CentBr1 and L17 probes, respectively. **(C,D)**, **(F,G)**, and **(I)** focusing on C4 were derived from **(B,E,H)**, respectively. 5S rDNA, L17 and CentBr1 were labeled with Alexa Fluor™ 488-5-UTP (green), and pWY96 was labeled with Texas Red-5-dCTP (red). The arrows indicated chromosome C4. Here, owing to the telomere-mediated truncation of chromosome C4, the weak signal of BAC clone KBrB072L17 was lost in C4 at the end of its long arm. Scale bar = 10 μm.

Previously, Xiong and Pires (2011) found 5S rDNA was located on the long arm of chromosome C4 near the centromere, a strong and a weak signals of BAC clone KBrB072L17 were located on chromosome C4 at the end of its short and long arms, respectively, and CentBr1 repeats could be visualized in *B. napus* chromosome C4 by multi-FISH (Figure 6A). These features of chromosome C4 were different from the remaining chromosomes in *B. napus* (Xiong and Pires, 2011). So, 5S rDNA, BAC clone KBrB072L17 and CentBr1 can be utilized as multi-FISH probes to display and confirm the truncation of chromosome C4 in the offspring of 86-1-75-10-74. In this study, 5S rDNA (Figures 6B–D), CentBr1 repeats (Figures 6E–G), and a strong signal for the BAC clone KBrB072L17 (Figures 6H,I) could be detected, while the weak signal for the BAC clone KBrB072L17 was lost (Figures 6H,I). These multi-FISH results demonstrated the minichromosome made by telomere-mediated chromosome truncation in C4 at the end of its long arm.

### Pollen Abortion of Transgenic Plants With Truncated Chromosomes

**Figure 7:**
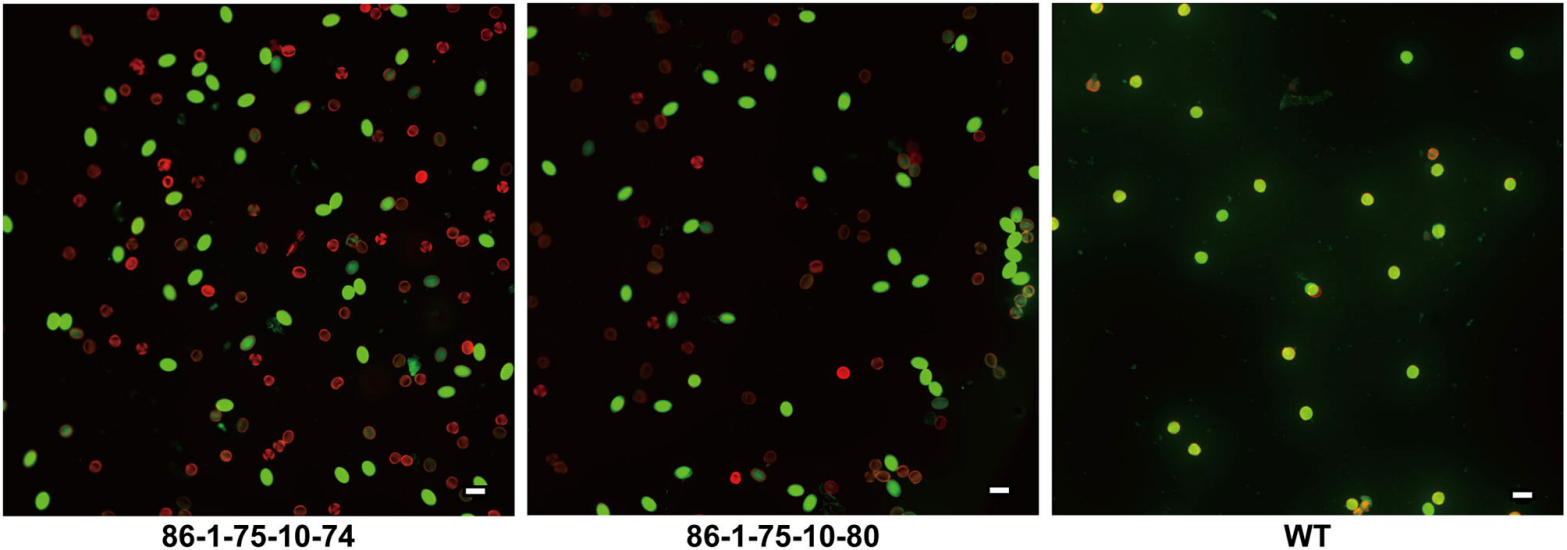
Detection of pollen fertility and activity by FDA-PI. The pollen of 86-1-75-10-74, 86-1-75-10-80, and WT were stained with 3,6-diacetoxyfluoran-propidium iodide (FDA-PI) to detect their fertility and activity. FDA (green) indicates active pollen; PI (red) indicates inactive pollen. The results showed that only ~50% of pollen of transgenic plants containing minichromosomes was active, while the active pollen of WT accounted for more than 90%. Bar = 50 μm.

Pollen abortion was observed in previous studies of plants with artificial minichromosomes owing to telomere-mediated chromosomal truncation (Yu et al., 2006; Yan et al., 2017). In this study, the pollen fertility and activity in transgenic plants and their offspring with truncated chromosomes were analyzed through 3,6-diacetoxyfluoran-propidium iodide (FDA-PI) staining. PI showing red fluorescence can only penetrate the cell membranes of dead cells, while FDA presenting green fluorescence can only permeate the cell membranes of living cells (Jones and Senft, 1985; Oliver et al., 2005). It was firstly observed that more than 90% of the pollen was abortive in T0 transgenic plants and no T1 progeny by selfing T0 transgenic plants with minichromosomes made by telomere-mediated chromosome truncation were obtained. The T0 transgenic plants with the minichromosomes were crossed with WT plants and 21 progeny lines were obtained. The pollen abortion of 86-1-75-10, one of the progeny lines, was detected by FDA-PI staining. The results showed that only ~50% of pollen containing artificial minichromosomes was abortive, while the wild type plants had more than 90% fertile pollen (Figure 7). It's inferred that the pollen abortion was caused by telomere-mediated chromosomal truncation due to the loss of genetic information in transgenic plants.

### Transmission of *B. napus* Minichromosomes

**Figure 8:**
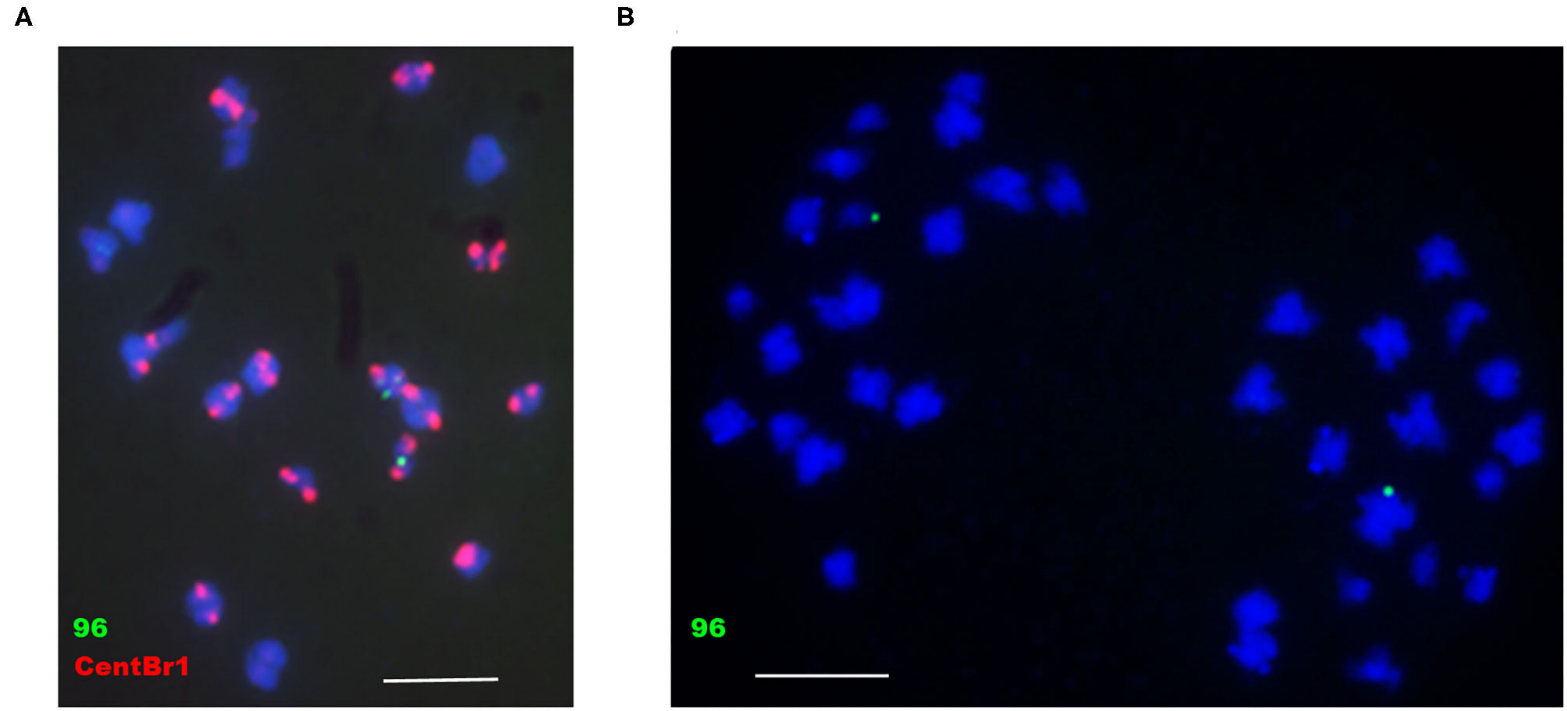
Analysis of the behavior of minichromosomes in meiotic metaphase II and anaphase II by FISH. **(A)** 86-1-75-10-2, meiotic metaphase II; **(B)** 86-1-75-7-3, meiotic anaphase II. pWY96, green; CentBr1, red; DAPI, blue. Scale bar = 10 μm.

To determine whether *B. napus* minichromosomes produced by the truncation of chromosomes were transmissible, 86-1-73 and 86-1-75 were selfed or hybridized with WT. T1 progenies were not successfully obtained. However, F1 progenies could be obtained with low seedset rate by hybridizing T0 transgenic plants with WT, in which whether T0 transgenic plants were used as the male or the female parent. To characterize the behavior of minichromosomes during transmission, the microspores of meiotic metaphase II and anaphase II from 86-1-75-10-2 and 86-1-75-7-3 with truncated C4, which were detected by PCR with truncated C4 primers, were probed by pWY96 with or without repetitive sequence probe CentBr1 (Xiong and Pires, 2011) (Figure 8). We found at least 10 cells behave as the same as Figure 8 in detected total 30 cells, and others behaver could not be confirmed due to poor hybridization signal. The results showed that the sister chromatids of minichromosomes could be separated and distributed normally as intact chromosomes during meiosis, which ensured normal transmission of minichromosomes.

### Chromosomal Recombination *via* a Site-Specific Recombination System

**Figure 9:**
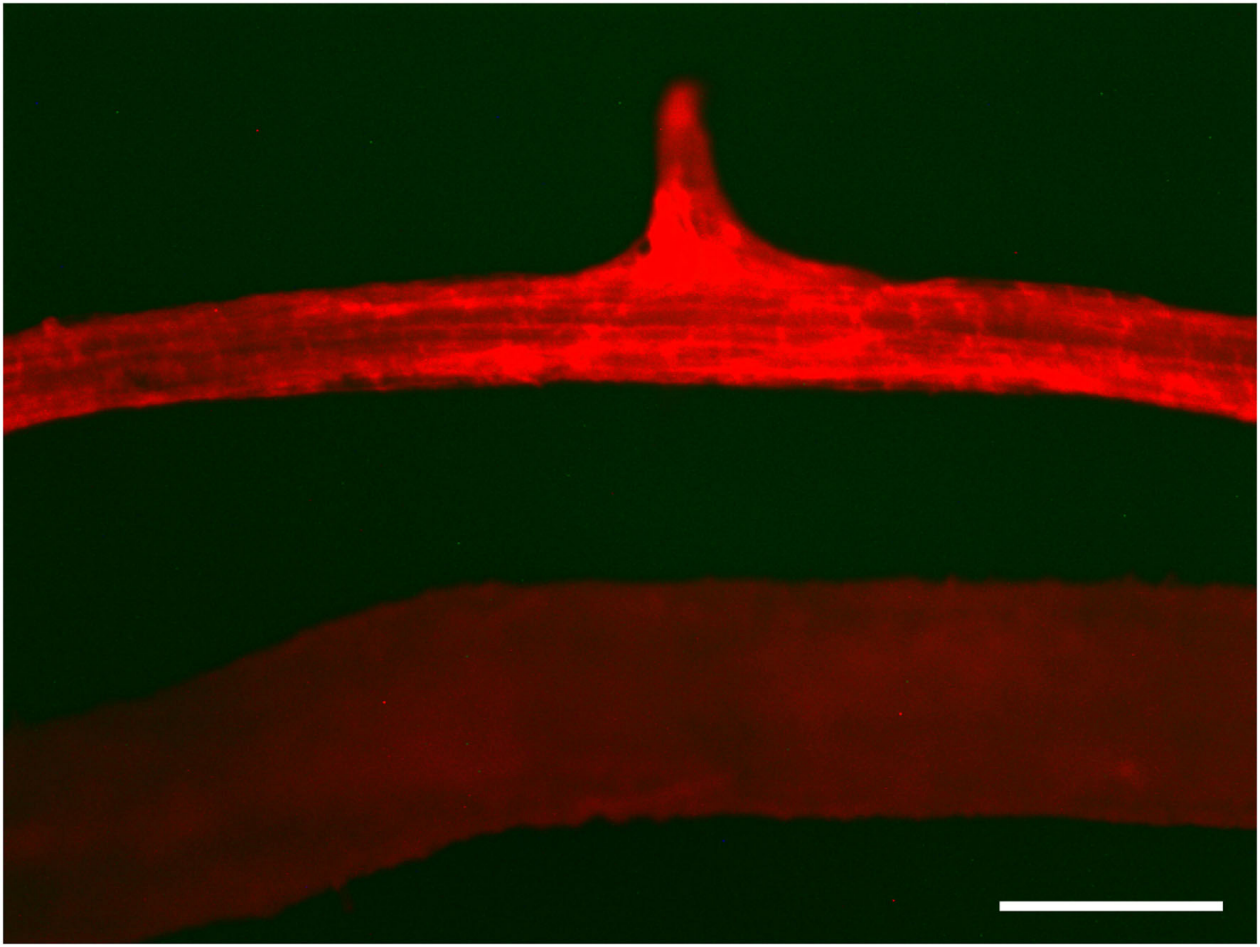
Cre/lox and FLP/FRT-mediated site-specific recombination. Red fluorescence was detected in the recombination of 1301-3-221-4-1 and 86-1-75-10-1 in which P35S in the pCre1301 transgenic plant was precisely inserted before DsRed and led to the expression of DsRed in engineered chromosomes by site-specific recombination in root tissue (upper) and the absence of red fluorescence protein in the 86-1-75-10-1 control (lower). DsRed fluorescence was observed by stereoscopic fluorescence microscopy. Bar = 500 μm.

Notably, minichromosomes are amenable to site-specific recombination manipulations, which determines whether artificial chromosomes can be employed as independent platforms for foreign gene expression. Site-specific recombination, a valuable tool for the removal of marker genes (Kerbach et al., 2005), gene targeting (Choi et al., 2000; Chawla et al., 2006) and gene conversion (Djukanovic et al., 2006), occurs in somatic cells, unlike genetic recombination that depends on meiotic pairing at meiosis. Site-specific recombination could be applied to add genes to the platforms. Yu et al. (2007a) also provided evidence of site-specific recombination using Cre. In the vector pWY86-1, a promoter-less lox71-DsRed cassette is present. In the vector pCre1301, the P35S-lox66-Cre-Tnos expression cassette was present. To detect the specific site insertion of foreign gene in the minichromosome, the red fluorescence signal of F1 plantlet, which was obtained by crossing between pWY86-1 transgenic plant 86-1-75-10-2 containing minichromosome and pCre1301 transgenic plant 1301-3-221-4-1, was observed with a stereoscopic fluorescence microscope (Figure 9). These results demonstrate that P35S in the pCre1301 transgenic plant was precisely inserted in front of DsRed and mediated the expression of DsRed in engineered chromosomes by a site-specific recombination system in *B. napus* and that the engineered minichromosome can be used as a platform for stacking foreign genes.

## Discussion

In this study, we succeeded in producing artificial minichromosomes in *B. napus* by introducing T-DNA constructs with telomere repeats mediated by *Agrobacterium*, in agreement with previous studies (Yu et al., 2006, 2007a; Nelson et al., 2011; Teo et al., 2011; Kapusi et al., 2012; Xu et al., 2012; Tada et al., 2015; Yan et al., 2017; Yuan et al., 2017). Using the methods described in the section Materials and Methods, pWY86-1 was inserted into the genome of *B. napus*, but there were up to six insert sites on at least five different chromosomes (Figures 1, 5), of which two inserts resulted in two truncated chromosomes, C2 and C4. Plants with multiple inserts could not set seeds, and offspring with truncated chromosomes could be obtained only by crossing pWY86-1 transgenic plants with wild-type plants and subsequently selfing (Figure 5). The crossing led to the separation of a single truncated chromosome from other inserts and truncated chromosomes (Table 2), and the truncated chromosomes can be transmitted to the next generation or different varieties of *B. napus* by additional crossing. Furthermore, we confirmed that the truncated minichromosome can be employed as a platform to receive foreign genes (Figure 9) in *B. napus*, in keeping with the findings of a previous report (Yan et al., 2017).

### Characterization of Truncated Chromosomes

According to previous reports, chromosome truncation events were mainly characterized through three methods: FISH, Southern blotting, and PETRA (Yu et al., 2006; Nelson et al., 2011; Teo et al., 2011; Kapusi et al., 2012; Xu et al., 2012; Yan et al., 2017; Yuan et al., 2017). Thus, we characterized chromosome truncation events using these three methods (Figures 2–4). However, because the chromosomes of *B. napus* are very small in size, FISH using pWY96 as the probe could not identify the chromosome that was truncated, except for the identification of insertion events. In fact, the FISH results did not show truncation events in the development of minichromosomes in *B. napus* reported by Yan et al. (2017). However, researchers have developed several useful probes to distinguish different chromosomes of *B. napus* by their different locations on chromosomes. For example, 5S rDNA was located on the long arm of C4 near the centromere, BAC clone KBrB072L17 was located in C4 at the end of its short and long arms, and CentBr1 repeats were visualized on the centromere of *B. napus* chromosomes, including C4 (Xiong and Pires, 2011; Xiong et al., 2011). We used these three probes and pWY96 to confirm the truncated chromosome C4 (Figure 6). Therefore, multi-FISH could be an efficient tool to identify chromosome truncation events in *B. napus*.

Southern blot analysis and PETRA can only confirm chromosome truncation events by detecting new telomere formations of different sizes (showing smear bands in Southern blotting and PETRA results) but cannot identify the precise truncation position of chromosomes. The precise truncation positions of chromosomes have rarely been reported. In this study, we resequenced the genome of transgenic plant 86-1-75 offspring and found two truncated chromosomes, C2 and C4 (Figure 5), except for three inserts without truncation. Furthermore, we designed insertion site-specific primers to confirm the chromosome truncation of C2 and C4 (Figure 5).

Chromosome walking was used to find the insert position of T-DNA in the development of minichromosomes in *B. napus* (Yan et al., 2017). A total of five T-DNA flanking sequences were obtained, but the results could not be directly utilized to confirm whether the truncated chromosome was present. Chromosome walking combined with PETRA was used to characterize the chromosome truncation events in the offspring of pWY86 transgenic plants by crossing them with *B. napus* Zhongyou 821 (Yan et al., 2017). However, the chromosome and the truncation site could still not be definitely determined.

### Transmission and Tracking of Minichromosomes

The stable transmission of minichromosomes is essential if minichromosomes are to be used as platforms for receiving foreign genes. To detect the transmission of minichromosomes between generations, FISH and PETRA methods were used (Yu et al., 2007a; Kapusi et al., 2012; Xu et al., 2012; Yan et al., 2017; Yuan et al., 2017). In diploid plants, minichromosomes resulted from telomere-mediated chromosomal truncation could be stable in callus but not transmissible in sexual reproduction, or plantlets containing them could not survive, while in tetraploid plants, they were transmissible in sexual reproduction but were inherited at rates lower than expected according to Mendelian rules (Yu et al., 2007a; Kapusi et al., 2012; Xu et al., 2012; Yan et al., 2017; Yuan et al., 2017). Yan et al. (2017) used T-DNA flanking sequence to detect the transmission of potential truncated chromosomes. However, the uncertainty of transmission could be increased if the truncated chromosome was not in the homozygous condition.

In this study, we confirmed that truncated chromosomes C2 and C4 can be tracked in different generations by insertion site-specific PCR (Figure 5C). The insertion site-specific PCR used for tracking minichromosome transmission is an efficient, simple, and accurate method that has not been reported previously. Furthermore, we observed the behavior of minichromosomes during meiosis (Figure 8). The results showed that the truncated chromosomes can normally pair and separate, which is the genetic basis for the stable inheritance of minichromosomes.

### FISH Techniques for Characterizing Chromosomes in *B. napus*

FISH is a very useful technique in molecular cytogenetics research. However, the FISH results are usually not clear enough due to their small size and the difficulty of preparing chromosome samples with high quality. In previous FISH work on chromosomes of *B. napus* (Xiong and Pires, 2011), chromosome samples were prepared using immature flower buds (~2 mm long) following the enzyme maceration method of Kato et al. (2004), and FISH was performed following the method of Kato et al. (2004) with slight modifications. In this study, we treated root tips and suitable flower buds using N_2_O. and FISH was performed using a probe directly labeled with Texas Red-5-dCTP (Perkin Elmer Life Sciences) or ChromaTide™ Alexa Fluor™ 488-5-UTP (Invitrogen) using nick translation as previously described (Kato et al., 2004). The chromosome images and FISH signals were significantly improved (Figures 6, 8). This method has been used in maize and wheat (Yu et al., 2006, 2007a; Yuan et al., 2017). In this study, it was confirmed that this method is suitable for FISH analysis of *B. napus* chromosomes.

### Transformation Technique in *B. napus*

The transformation technique for *B. napus* is mature. Using the hypocotyl as the explant, the rate of transformation was high using screening chemicals kanamycin or hygromycin (Radke et al., 1988; Moloney et al., 1989; Damgaard et al., 1997; Zhang et al., 2005; Bhalla and Singh, 2008). In this study, because we used the vector pWY86 with the *Bar* selection marker gene to perform the transformation, the rate of transformation was low and multiple copies were usually inserted into the genome of *B. napus* (Figure 2; Yan et al., 2017). Multicopy insertions would lead to developmental problems in transgenic plants due to multichromosome truncations, such as pollen sterility (as in this study), and difficulty in the detecting chromosome truncation events. Therefore, in future studies on the development of *B. napus* artificial minichromosomes, the selection marker gene *NPT* or *HPT* should be chosen.

### Receptors of Artificial Minichromosomes

In previous work, plants with truncated chromosomes were usually sterile or dead, and minichromosomes could not be transmitted to the next generation unless recovered in a tetraploid (Yu et al., 2006), although a very small minichromosome derived from an A chromosome was recovered along with the diploid complement of chromosomes in maize (Gaeta et al., 2013). To overcome lethality induced by chromosomal truncations, some strategies can be used. In maize, engineered minichromosomes were constructed successfully from supernumerary B chromosomes attained from 12 to 39% through the male parent by using telomere-mediated chromosomal truncation (Yu et al., 2007a). There are almost no essential genes in B chromosomes; thus, the truncation of B chromosomes will not cause developmental or transmission problems, as A chromosomes do (Yu et al., 2007a). In barley, constructs containing telomeres were transformed into tetraploids and diploids. However, chromosome truncation was found only in tetraploid plants, and the transmissibility of minichromosomes was lower than that expected according to Mendelian rules, which indicated that the transmission of minichromosomes may be unstable even in polyploid plants (Kapusi et al., 2012). In wheat, a high frequency and stable transmission of chromosomal truncations, which produced minichromosomes, was successfully performed (Yuan et al., 2017).

In this study and in the study of Yan et al. (2017) *B. napus*, an allotetraploid, could withstand multiple insertions and at least two chromosome truncations. Plants with two truncated chromosomes can survive, and the truncated chromosome can be transmitted to subsequent generations. Therefore, polyploid plants, such as wheat, *B. napus*, and tetraploid barley, should be good receptors for truncated chromosomes. In addition, addition lines may also be good materials for constructing artificial minichromosomes through telomere-mediated chromosomal truncation. Previous studies have obtained various monosomic and disomic addition lines of *Brassica napus* by hybridization and backcrossing (Jahier et al., 1989; Chevre et al., 1991; Struss et al., 1991; Snowdon et al., 2000; Wang et al., 2006; Akaba et al., 2009; Wei et al., 2010; Kang et al., 2014), and addition chromosomes are not essential for plants. Thus, truncation of addition chromosomes will not affect the growth and development of plants and could increase the transmission rate of minichromosomes (Xu et al., 2012).

### Perspective for Constructing Artificial Minichromosomes by Telomere-Mediated Chromosomal Truncation

To date, chromosome truncation has been randomly completed due to random insertion of telomere sequences. The truncated chromosomes and the truncation sites are not precisely designed. Plants with truncated chromosomes usually grow abnormally or die. Homologous recombination is a research hot point in plant genetic engineering and would be the most promising method to avoid the problem of random insertions of foreign genes. Significant progress has been achieved in homologous recombination coupled with TALENs or CRISPR/Cas9 in several plants, including *ANT1* gene overexpression by inserting the *35S* promoter in tomato (Cermak et al., 2015), replacement of the *ARGOS8* promoter with a native *GOS2* promoter in maize to improve yield under drought stress maize (Shi et al., 2017), and engineering herbicide-resistant rice and maize plants through CRISPR/Cas9-mediated homologous recombination of acetolactate synthase (Svitashev et al., 2015; Sun et al., 2016). Future artificial minichromosomes should be predesigned, including the selected chromosome and the truncation position. This step could be achieved by bioinformatics analysis of target receptor plants and highly efficient homologous recombination techniques with TALENs or CRISPR/Cas9. Well-designed artificial minichromosomes would be a new tool for studying the structure and function of chromosomes and an efficient platform for next-generation gene engineering and could be easily identified and tracked.

In conclusion, in this study, C2 and C4 artificial minichromosomes were developed in *B. napus* using telomere-mediated chromosomal truncation. The truncated chromosomes and the truncation sites were precisely characterized by transgenic plant genome resequencing, insertion site-specific PCR and multicolor FISH. The truncated artificial minichromosomes can be transmitted to the next generation and tracked by simple insertion site-specific PCR. The truncated minichromosomes can receive foreign P35S which could drive DsRed expression. This study provided two artificial minichromosomes, C2 and C4, and showed the new approaches to precisely identify the truncated chromosomes and the truncation sites and easily track minichromosome transmission. Finally, a development strategy for obtaining ideal artificial minichromosomes in the future was proposed.

## Data Availability Statement

The raw data supporting the conclusions of this article will be made available by the authors, without undue reservation.

## Author Contributions

ZH, CF, and XY initiated and designed the experiments. XY and YZ performed the research and wrote the paper. YC, CF, JW, and ZH discussed the results and modified the manuscript. RW helped advise graduate research, interpreted and discussed data, and revised the manuscript. All authors have read and approved the manuscript.

## Funding

This work was funded by the Strategic Priority Research Program of the Chinese Academy of Sciences (Grant No. XDA24030502), the Key Research and Development Project from Department of Science and Technology of China (Grant Nos. 2016YFD0100506 and 2016YFD0102003-10), the National Transgenic Research Projects (Grant Nos. 2014ZX0801006B and 2009ZX08010-003B), and Natural Science Foundation of China (Grant Nos. 31271755 and 32072095).

## Conflict of Interest

The authors declare that the research was conducted in the absence of any commercial or financial relationships that could be construed as a potential conflict of interest.

## Publisher's Note

All claims expressed in this article are solely those of the authors and do not necessarily represent those of their affiliated organizations, or those of the publisher, the editors and the reviewers. Any product that may be evaluated in this article, or claim that may be made by its manufacturer, is not guaranteed or endorsed by the publisher.

## Supplementary Material

Supplementary Figure 1 Verification of pWY86-1 and pCre1301 transgenes by PCR. **(A)** PCR analysis of pWY86-1 transformants. Lane 1, WT, negative control; lanes 2, 5, 6, and 8, negative transformants of pWY86-1, marked as 86-1-15, 86-1-80, 86-1-87, and 86-1-94, respectively; lanes 3, 4, and 7, positive transformants of pWY86-1, marked as 86-1-73, 86-1-75, and 86-1-90, respectively; lane 9, pWY86-1, positive control; lane 10, Trans2K Plus II DNA Marker. The primers were FLP_F (5′-ATAACGGAACAGCAATCAAG-3′) and FLP_R (5′-GTAGGATGAAAGGTAGTCTAG-3′). **(B)** PCR analysis of pCre1301 transformants. Lane 1, 1301-1; Lane 2, 1301-2; Lane 3, 1301-3; Lane 4, 1301-4; Lane 5, pCre1301; Lane 6, WT. The primers were GUS_F (5′-GATGTCACGCCGTATGTTATTGCC-3′), GUS_R (5′-CGTAATAACGGTTCAGGCACAGC-3′), HPT_F (5′-ATGAAAAAGCCTGAACTCACCGC-3′), and HPT_R (5′-CTATTTCTTTGCCCTCGGACGAGT-3′).

Supplementary Table 1 All primers used in this study.
