# Supplementary figures and images for: Precise Characterization and Tracking of Stably Inherited Artificial Minichromosomes Made by Telomere-Mediated Chromosome Truncation in Brassica napus
Source: Front Plant Sci. 2021 Oct 4;12:743792. doi: 10.3389/fpls.2021.743792 (PMC8521072; doi:10.3389/fpls.2021.743792)

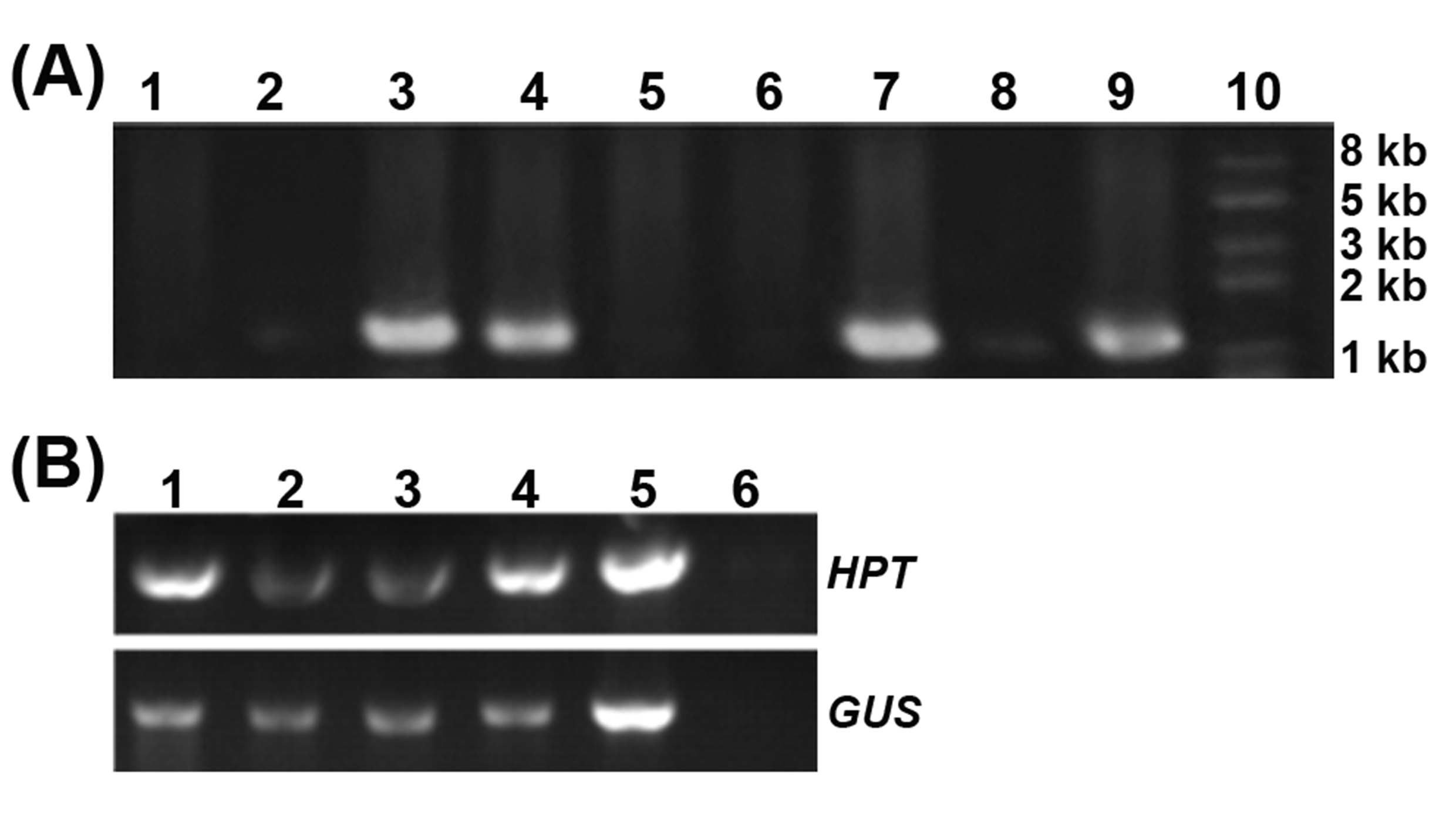

Supplement: Supplementary Figure 1 — Verification of pWY86-1 and pCre1301 transgenes by PCR. (A) PCR analysis of pWY86-1 transformants. Lane 1, WT, negative control; lanes 2, 5, 6, and 8, negative transformants of pWY86-1, marked as 86-1-15, 86-1-80, 86-1-87, and 86-1-94, respectively; lanes 3, 4, and 7, positive transformants of pWY86-1, marked as 86-1-73, 86-1-75, and 86-1-90, respectively; lane 9, pWY86-1, positive control; lane 10, Trans2K Plus II DNA Marker. The primers were FLP_F (5′-ATAACGGAACAGCAATCAAG-3′) and FLP_R (5′-GTAGGATGAAAGGTAGTCTAG-3′). (B) PCR analysis of pCre1301 transformants. Lane 1, 1301-1; Lane 2, 1301-2; Lane 3, 1301-3; Lane 4, 1301-4; Lane 5, pCre1301; Lane 6, WT. The primers were GUS_F (5′-GATGTCACGCCGTATGTTATTGCC-3′), GUS_R (5′-CGTAATAACGGTTCAGGCACAGC-3′), HPT_F (5′-ATGAAAAAGCCTGAACTCACCGC-3′), and HPT_R (5′-CTATTTCTTTGCCCTCGGACGAGT-3′). [file Image_1.TIF]
