# Supplementary material for: Precise Characterization and Tracking of Stably Inherited Artificial Minichromosomes Made by Telomere-Mediated Chromosome Truncation in Brassica napus
Source: Front Plant Sci. 2021 Oct 4;12:743792. doi: 10.3389/fpls.2021.743792 (PMC8521072; doi:10.3389/fpls.2021.743792)
Supplement: Supplementary Table 1 — All primers used in this study. [file Table_1.docx]

**Table S1. All primers used in this study**

| Primer | Sequence (5' - 3') | Annotation |
| --- | --- | --- |
| FLP_F | ATAACGGAACAGCAATCAAG | 1,115 bp; Detecting pWY86-1 transformants; |
| FLP_R | GTAGGATGAAAGGTAGTCTAG |  |
|  |  |  |
| GUS_F | GATGTCACGCCGTATGTTATTGCC | 1,057 bp; Detecting pCre1301 transformants |
| GUS_R | CGTAATAACGGTTCAGGCACAGC |  |
|  |  |  |
| HPT_F | ATGAAAAAGCCTGAACTCACCGC | 1,026 bp; Detecting pCre1301 transformants |
| HPT_R | CTATTTCTTTGCCCTCGGACGAGT |  |
|  |  |  |
| PETRA-T | CTCTAGACTGTGAGACTTGGACTACCCTAAACCCT | PETRA; P4 for identifying the insertion or the truncation on chromosomes |
| PETRA-A | CTCTAGACTGTGAGACTTGGACTAC |  |
| p1 | TACGTCTAGATCTGGCGCGC |  |
| p4 | ATAATAGGATCCCCGATCGTT |  |
|  |  |  |
| CentBr1 F | GAATAGCACAGCTTCATCGTCGTTCC | Cloning the CentBr1 for probe |
| CentBr1 R | CTGGGAAACTGTAATCACCTGATCTGAAA |  |
|  |  |  |
| A1_F | GCTAATGTGCTTAGCTTGCTGTATC | Identifying the insertion on chromosome A1 |
| A1_R | CAACTTTCTGATTCATGTCGGTGAAC |  |
|  |  |  |
| A8_F | GCGAACGCGGTCACAGTT | Identifying the insertion on chromosome A8 |
| A8_R | TGGTTGAGGGATGAAACGGC |  |
|  |  |  |
| C1_F | CGGGAGCTTTGCACTAGAAATAGT | Identifying the insertion on chromosome C1 |
| C1_R | CTCTCATGTTCACCACAGACTCCA |  |
|  |  |  |
| C2_F | GATTTCCACTAAATTCAACCGTGCTC | Identifying the insertion and the truncation on chromosome C2 |
| C2_R | CTGACATCCGTGTTGTGAAGGC |  |
|  |  |  |
| C4_F | CTGAAACCTAATCTGATCCGAACCG | Identifying the insertion and the truncation on chromosome C4 |
| C4_R | CAAAGCCCAATTTCAAACATCAACT |  |
|  |  |  |
| Blp_F | ATGAGCCCAGAACGACGCC | Identifying the insertion or the truncation on chromosomes |
